# Nutritional Composition of Gluten-Free Labelled Foods in the Slovenian Food Supply

**DOI:** 10.3390/ijerph17218239

**Published:** 2020-11-07

**Authors:** Živa Lavriša, Maša Hribar, Anita Kušar, Katja Žmitek, Igor Pravst

**Affiliations:** 1 Nutrition Institute, Tržaška cesta 40, SI-1000 Ljubljana, Slovenia; masa.hribar@nutris.org (M.H.); anita.kusar@nutris.org (A.K.); katja.zmitek@nutris.org (K.Ž.); igor.pravst@nutris.org (I.P.); 2 Biotechnical Faculty, University of Ljubljana, Jamnikarjeva ulica 101, SI-1000 Ljubljana, Slovenia; 3 VIST–Higher School of Applied Sciences, Gerbičeva ulica 51a, SI-1000 Ljubljana, Slovenia

**Keywords:** gluten-free foods, nutritional composition, food labelling, food supply

## Abstract

The market of gluten-free (GF) foods has been expanding in recent years. GF foods are consumed not only by those with medical predispositions for avoiding gluten, but also by a specific segment of consumers, searching for “healthier” food choices. For these, such practices can present a serious limitation in the variability of food choices. Considering that GF foods are commonly perceived as healthier alternatives, there is a lack of knowledge on the nutritional profile and content of specific nutrients of GF-labelled foods compared to general food supply. A comparison of nutritional composition of GF/non-GF packed foods in the Slovenian food supply was conducted. The nutrient profiling scoring criterion (NPSC) and content of specific nutrients/energy was compared between GF-labelled and regular foods. The highest proportion of GF-labelled products were found in food categories, which typically do not contain gluten (Cheese imitates, Milk imitates, Yoghurt imitates, Canned fish and seafood and Processed meat). Significant differences in the nutrient profile between GF-labelled and regular products were found in Cakes, muffins and pastry, Crisps and snacks, Desserts and Milk imitates. GF-labelled foods often had lower protein and sugar content. Energy value was comparable in most categories and no significant differences in salt content were found, compared to non-GF products. In conclusion, GF-labelled foods will unlikely bring health benefits to those who are not medically required to follow GF diet. Public health initiatives should aim towards promotion of consuming non-processed foods and provision of reliable information about who is required to consume GF foods.

## 1. Introduction

Gluten is a protein found in certain cereals. Although harmless to most people, some individuals must avoid it due to medical reasons. Celiac disease (CD) is the most important disease where patients must avoid gluten. It is a systemic immune-mediated disorder, which causes permanent inflammatory enteropathy in genetically susceptible individuals, where gluten causes damage to the villi of the small intestine, which also results in malabsorption of nutrients [1]. It was estimated that approximately 1% of the world population suffers from this inheritable, autoimmune disease and together with those with other gluten-related disorders sums up to about 5% of the world population [2]. People diagnosed with CD are required to follow a lifelong gluten-free diet which is the only possible therapy for CD patients. Some people with functional gastrointestinal disorders may feel more comfortable on a GF diet but this is often in part due to the reduction of fermentable monosaccharides, oligosaccharides, disaccharides and polyols (FODMAP) which can occur in GF products [3]. However, CD patients are, besides some non-celiac gluten sensitive patients, the only ones who have the medical need to strictly follow a GF diet. In recent years, gluten-free (GF) diets have been receiving a lot of attention in the public discussions. There is growing public perception that GF foods are beneficial even for people without disease which require following such restrictive diets [4], therefore in the last decade adherence to GF diets has become popular in the general population as well [5]. Many consumers perceive GF foods as healthier than their gluten containing counterparts [6,7]. In some countries, reported prevalence rates of adherence to a GF diet are up to 6%. The main motivators for such a diet are believing that such a diet is healthier and helps with weight control [8]. Such beliefs led to a significant increase in demand of such products and the GF food market is still expected to grow. This trend is particularly notable in Western countries, where sales of GF products are already reaching several billion US dollars per year and are still on the rise [9].

Many gluten containing grains, such as wheat, barley and rye, are good sources of different nutrients and are contained in many widely consumed staple foods such as bread, pasta and breakfast cereals. These are particularly nutritious if consumed in whole grain products, rich in dietary fibre, which is lacking in Western diets. Excluding these grains from the everyday diet could have an impact on nutrient intake of individuals committed to a GF diet [10,11]. The nutritional composition of GF products is especially important for individuals who must follow a GF diet due to health reasons, as consuming GF products is not their personal choice, but a medical need. The poor nutritional composition of such products can have a long-term impact on the health of individuals bound to a GF diet. Studies report that dietary inadequacies are common among people, adherent to a GF diet. It was found that their intake of dietary fibre was often inadequate, along with lower intakes of certain micronutrients, e.g., vitamin A, B vitamins, calcium, iron and zinc. This could cause various health problems, from low mineral bone density to micronutrient deficiencies and other diseases and conditions [11,12]. Due to these reasons, CD patients who must adhere to a GF diet could suffer from health risks if their nutritional intake is not properly balanced. Considering the diet of CD patients and the increased popularity of GF products in the population there is need for evaluation of the nutritional composition of such foods. This is particularly important when it comes to non-staple, processed foods. Evidence from previous studies suggests that GF products compared to regular gluten containing products from the same food categories might not present a healthier alternative to people without gluten-related disorders [13,14]. GF products were often less nutritious and had less protein and dietary fibre, and sometimes also higher amounts of fat, saturated fat, sugar and salt, in comparison to regular products [15,16]. However, these observations could not be generalised, also because of the notable difference between various food categories. It should be noted that GF products are available even among food categories such as sweets, biscuits and snacks which are high in undesirable nutrients such as sugar, saturated fats and salt. Usually, GF alternatives in these food categories cannot be considered as healthy alternatives, and do not have improved nutritional composition in comparison with their gluten containing counterparts [17,18]. Another concern is that GF foods can be marketed as premium products and are sold at a higher price [14]. This can further contribute to the already established public opinion that such foods may be of higher quality and even healthier [14,19], even though this might not be the case.

The aim of the present study is to investigate the availability of GF prepacked foods in the food supply, and particularly to evaluate the nutritional quality of GF-labelled food products in comparison with products not marketed as GF. Nutritional quality was assessed for whole products using the nutrient profiling scoring criterion (NPSC), and with consideration of the energy content and specific nutrients.

## 2. Materials and Methods

### 2.1. Study Design and Data Collection

The source of data on nutritional composition and labelling information was the Slovenian branded foods dataset (CLAS 2015, Composition and Labelling Information System), which contains prepacked foods in the Slovenian food supply. The sample, methodology and data quality control have been described previously [20]. In brief, CLAS food monitoring is done as a cross-sectional study. To ensure national representativeness of the sample, data were collected in five grocery stores of retailers with the largest national market shares in Slovenia. This included two mega markets (Mercator centre, Interspar), two supermarkets (Spar, Mercator) and a discount market (Hofer). All prepacked food products with a unique European Article Number (EAN) barcode were photographed systematically and recorded in the online CLAS database [21]. Data on nutritional composition and other information from food labels were then extracted from photographs. For each product, data on energy value and content of saturated fats, protein, sugar, fibre and salt were collected. The content of fruit, vegetables and nuts in each product was also recorded. Only products with labelled nutritional composition were included in the study. For the purpose of nutrient profiling, in products where nutritional composition was labelled but only specific nutrients were missing (mostly for the content of dietary fibre), the missing nutritional values were supplemented from a food composition database, using the previously described method [22]. For this study, all food labels were carefully checked for any brands or claims referring to being gluten free (GF). The dataset contained a total of 10,674 products. A sample for the present study included 8167 products with available nutritional composition, which were subject to nutrient profiling and comparison of energy value and content of specific nutrients. We compared energy, sugar, salt and protein content of GF and regular products in selected food categories and subcategories which contained at least 30 different GF-labelled products. The following few additional categories (with less than 30 different GF-labelled products) were also included due to their relevancy regarding gluten ingredients: Cakes, muffins and pastry, Bread, Breakfast cereals and Dry pasta.

### 2.2. Food Categorization and Nutrient Profiling

All products were categorized into specific food categories, according to classification which was developed as a part of the Global Food Monitoring Initiative by Dunford et al. [23]. Some minor modifications to categories were made due to European market specifics, as previously described [20]. The final sample included 13 parent categories, 43 categories and 82 subcategories. Data for all parent categories, categories and subcategories are listed in Supplementary Table S1.

Each product was evaluated according to the nutrient profiling scoring criterion (NPSC) which was developed by Food Standards Australia New Zealand (FSANZ) [24]. The NPSC is a nutrient profiling system used in Australia and New Zealand to determine whether a certain food is suitable to make a health claim, based on its nutrient profile determined from nutritional composition. The nutrient profile refers to raw foods, but if a food requires preparation, the point calculations must be based on the food prepared according to manufacturer’s instructions. NPSC divides foods into three categories Category 1–beverages; Category 2–any food other than those included in Category 1 or 3; and Category 3–cheese or processed cheese (with calcium content >320 mg/100 g), edible oil, edible oil spread, margarine, butter. These food categories are used to determine how some of the scoring points are allocated in the calculations. After determining food categories, baseline points must be calculated. For this, it is necessary to know the energy content and content of saturated fat, sugar and sodium in 100 g or 100 mL of the food. Furthermore, modifying points need to be calculated, which consist of fruit and vegetable (V) points, protein (P) points and fibre (F) points. The final score is then calculated by subtracting the modifying points from the baseline points. For each food category (1, 2 or 3), the threshold limit for final score points are set. Based on these points, foods were classified either as “Healthier” or “less healthy” [25].

For statistical evaluation of differences between nutrient profiling outcomes between GF-labelled and regular products, food categories and subcategories with more than 15 GF-labelled products were selected.

### 2.3. Data Processing and Statistical Analyses

Food composition and labelling data were processed using Microsoft SQL Server Management Studio 13.0, Microsoft Analysis Services Client Tools 13.0, Microsoft Data Access Components (MDAC) 10.0, Microsoft Excel 16.0 (Microsoft, Redmond, Washington, DC, USA) and the Composition and Labelling Information System (CLAS) (Nutrition Institute, Ljubljana, Slovenia). Statistical analyses were conducted using STATA version 15.1 (StataCorp LLC, Coledge Station, TX, USA).

Contents of energy and nutrients in selected food (sub)categories were presented as means. Standard deviations and differences in the mean content of selected nutrients were calculated. A comparison of means between GF-labelled and regular products was performed using a t-test for two independent samples. A chi-square test was used to assess differences in nutrient profiling outcome between products labelled as “GF” and those without such labelling. Differences were considered statistically significant at *p* < 0.005.

## 3. Results

In almost all food categories, the majority of foods were not labelled as GF (Supplementary Table S1). The exception was Cheese imitates, which had only two products, both labelled GF. The highest number of GF-labelled products was in the Dairy parent category (*n* = 123), followed by food categories which include products made of cereal: Bread and bakery products (*n* = 84) and Cereal and cereal products (*n* = 72). The parent category where GF-labelled foods were also common, was Meat and meat products (*n* = 68). When looking into the proportions, the food categories with the highest proportion of GF-labelled products were from the Dairy parent category: Cheese imitates (100%), Milk imitates (65%), and Yoghurt imitates (42%). Cheese, milk and yoghurt imitates are products that are typically consumed in same way as their dairy counterparts, but are plant based and could therefore potentially contain gluten. GF-labelled products were also quite common among Fish and fish products: Canned fish and seafood (42%), Fish spreads (19%), Sauces and spreads: Spreads (19%), and Processed meat and derivatives: Processed meat (16%).

**Figure 1:**
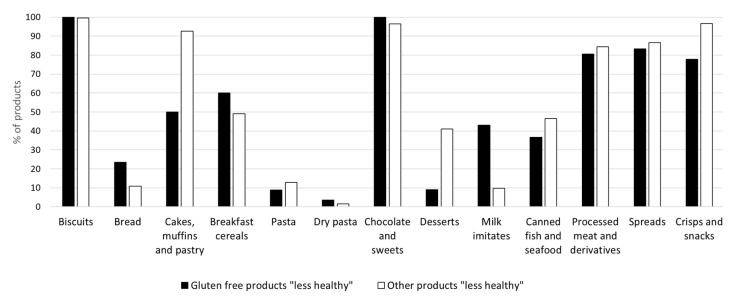
Proportion of products scored as “less healthy” (by the nutrient profiling scoring criterion (NPSC) in specific food categories for products labelled as gluten-free (■black bars) and for products without such labelling (□white bars).

Figure 1 shows the proportion of “less healthy” products in selected food categories and subcategories among GF-labelled and other products (data for complete dataset are provided in Supplementary Table S1). Food categories with significant differences (*p* < 0.01) between “less healthy” GF-labelled and other products were Cakes, muffins and pastry, Crisps and snacks and Desserts—with a higher proportion of regular products which scored as “less healthy”, while in Milk imitates a higher proportion of GF-labelled products scored as “less healthy”. A higher proportion of “less healthy” GF-labelled products was found also in the Bread subcategory, Breakfast cereals, Dry pasta and Chocolate and sweets, although these differences were not statistically significant. A notably higher proportion of “less healthy” GF products was driven by lower protein content in Breakfast cereals and Milk imitates, and by higher sugar content in Milk imitates, although in neither of these two subcategories were differences between nutrients in GF-labelled and regular foods significant.

We also conducted a comparison in the mean content of energy and certain nutrients between GF-labelled and regular products, focusing on food categories with the highest numbers of GF-labelled products (Table 1 and Table 2). The results revealed significant differences in content of specific nutrients between GF-labelled and regular products for Dry pasta, Cakes, muffins and pastry, and Canned fish and seafood. In general, GF-labelled products had lower mean levels of protein, except in Cakes, muffins and pastry and Sauces and spreads, but in both categories the difference in the mean was non-significant and was less than 1 g/100 g. There were no significant differences in mean salt content between GF-labelled and regular foods in any of the selected categories and subcategories; in general, the salt content of GF-labelled vs. regular foods was quite similar.

Among widely and often consumed staple foods, Bread category, which, besides bread, includes also crisp bread, dough and tortillas, had similar mean content of nutrients comparing GF-labelled and regular products, except for protein, which was lower in GF-labelled breads (Δ_protein_ 9.7 g/100 g vs. 5 g/100 g, *p* < 0.01). Lower energy value of GF-labelled foods in this category was observed, but difference was not significant (ΔE 108.6 kJ, *p* = 0.1). As mentioned earlier, GF-labelled Dry pasta had higher proportion of “less healthy” products, compared to regular products. Although it had significantly lower means of energy value (ΔE 151.4 kJ, *p* < 0.01) and sugar (Δ_sugar_ 2.2 g, *p* < 0.01), GF-labelled Dry pasta products had more than twice as less the value of protein compared to regular Dry pasta (Δ_protein_ 6.6 g, *p* < 0.01).

GF-labelled non-staple, cereal containing foods from Cakes, muffins, and pastry and Biscuits categories had lower mean content of undesirable nutrients, such as sugar. However, GF-labelled Biscuits had significantly lower protein content compared to regular products (*p* < 0.01). Anyway, both GF and regular products from these two categories mostly included “less healthy” products.

Among food categories with non-cereal based ingredients (which can contain gluten or gluten containing ingredients), presented in Table 2, processed meat products and canned fish products were those commonly labelled as GF. In the Canned fish and seafood category, GF-labelled products contained more sugar (Δ_sugar_ 1.1 g, *p* < 0.01) and less protein (Δ_protein_ 2.0 g, *p* < 0.01). GF-labelled Processed meat and derivatives had lower mean energy values compared to regular products (ΔE 138.7 kJ, *p* = 0.04) and, besides Dry pasta, this was the only category with significant differences in mean energy value between GF and regular products.

## 4. Discussion

The present study is the first one evaluating the presence of GF-labelled prepacked food products in the Slovenian food supply and their nutritional composition in comparison to regular foods. Results of the present study show that GF labelling is the most common among foods that are in fact not typical sources of gluten. For example, in the subcategory of Milk imitates, 65% of products were labelled as GF, although many of them were made of GF cereals or even nuts (e.g., rice milk, soy milk, almond milk). Over 10% of products in Fish and fish products and Meat and meat products’ parent categories also carry the GF label, which was not seen to such an extent in other non-cereal based categories. Although these food categories were among those with the highest proportion of GF-labelled products within our sample, the proportion was still much lower than was it found for example in an Australian evaluation of supermarket products, where 87% of processed meat was labelled as GF [13]. Gluten is widely used in meat and fish products due to its binding properties. It successfully binds fat and water, and therefore it is useful for binding pieces of meat to produce meat products with better slicing and shaping characteristics [26]. Meat and fish GF- labelled products from our sample contained less protein, suggesting lower a meat content of these products. Additionally, GF-labelled Canned fish and seafood products also had higher sugar content (*p* = 0.004), compared to foods not labelled as GF.

Gluten is not only known as constituent of certain grains; in isolated form, it is also widely used in food processing due to a wide range of functional properties. In the bakery industry, gluten is mostly known for its dough leavening properties [26]. It is a protein that builds dough structure which is necessary for achieving the desired results of baked goods. It is also used for fortification of flours with lower protein content, which improves the flour quality. In bakery products which do not contain gluten or gluten containing flours, food manufacturers are using a variety of mixtures of starches, hydrocolloids, gums and other ingredients to achieve desirable product properties without using gluten-containing ingredients [27]. The cost of using gluten is usually lower than the cost of some other proteins, found in e.g., soy or milk, and this can be an important factor for the extensive use of gluten in the food industry [26]. Study results indicate some differences in the nutritional profile and content of specific nutrients between GF-labelled and regular foods, but these differences cannot be generalized. Although in some selected food (sub)categories there was a slightly lower proportion of “less healthy” GF-labelled foods, the nutrient profile of processed GF-labelled foods was generally poor; the sample mostly included processed foods, classified as “less healthy” according to the NPSC. This finding is in line with other studies, which showed that the nutritional composition of GF foods is usually not superior to regular products [13,17,18,28]. It is interesting that in Cakes, muffins and pastry, which are generally considered as foods with poorer nutritional composition, the proportion of “less healthy” products among GF-labelled foods was significantly lower (*p* < 0.01). Interestingly, this category was significantly (*p* < 0.01) lower in sugar content, which supported their overall nutritional quality rating. Although non-significant, there were more “less healthy” products in the GF-labelled Bread subcategory which could be concerning for those regularly or even exclusively consuming it, as such staple foods are often utilised on daily basis. It should be noted that our sample only includes prepacked bread. GF bread is often available as prepacked to enable highlighting of the GF properties, and to prevent contamination with gluten from other products which are often available at the same facilities. Such bread is likely bought especially by those who need to follow a restrictive GF diet. An important insight is also that GF-labelled foods were generally lower in protein, which is a similar finding to those in previously published studies [13,14,18,29,30]. The fact that gluten is a protein might contribute to this finding, as gluten containing ingredients must be replaced with those without gluten, and this can potentially lower protein content in foods. From a nutritional point of view, this might not be a huge issue, considering that cereal based foods are usually not the main contributors of protein in the diet. Although it is still important which substitute ingredients are used instead of gluten. GF ingredients which are used as gluten substitutes are usually carbohydrate-based ingredients such as maize or potato starch and refined GF flours, which are poor in micronutrients and fibre [31]. In order to improve the nutritional composition of GF foods, some pseudo cereals, such as amaranth, quinoa and buckwheat were proposed [32]. These are rich in desirable nutrients, such as protein, fibre, vitamins and minerals, but the use of such ingredients influences both sensorial properties and manufacturing costs of the products. Studies show that the intake of fibre in a GF diet is often inadequate and a higher intake of fibre-rich whole grains is recommended [12,33]. Another nutrient important from the public health perspective is sodium. Looking into the nutritional composition of products in our dataset, we found no significant differences in sodium/salt content between GF-labelled and regular products, although some studies report higher salt content in GF foods [17,34]. However, salt content was still high in some categories, for example in Processed meat and derivatives and Sauces and spreads, although not significantly differing from regular products. Energy value was significantly lower in the subcategory of GF-labelled Dry pasta (*p* < 0.01). This could be due to the different grain and/or cereal ingredients of GF-labelled vs. regular products in this subcategory, which affected the energy content of these products. A similar reason probably contributed to the lower energy value in GF-labelled Breads, although the difference was not statistically significant (*p* = 0.1).

According to the European Union (EU) “GF” food labelling regulations, such a claim can be only used on foods which do not contain more than 20 mg/kg of gluten [35]. However, general EU regulation on the provision of food information to consumers also states that food information must not be misleading, especially “by suggesting that the food possesses special characteristics when in fact all similar foods possess such characteristics, in particular by specifically emphasizing the presence or absence of certain ingredients and/or nutrients” [36]. From this point of view, there is a dilemma, whether GF labelling is justified on products that typically do not containing gluten, as other products from the same category have the same characteristics (even if not labelled as GF). On the other hand, the threshold limit for gluten content in foods is relatively low and cross-contamination is also possible, particularly in manufacturing facilities that process gluten containing products as well. In such cases, it is questionable if food manufacturers are aware that the 20 mg/kg limit for gluten presence in GF-labelled foods can be exceeded when production is not carefully handled. GF labelling is not justified on such a product. This topic is not extensively investigated, there is evidence that, although usually in small proportions, exceeding gluten limits can occur. This can happen in foods not labelled as GF but manufactured using ingredients where gluten content is not expected, and also in foods specifically labelled as GF [37,38,39,40]. Many GF-labelled foods are considered processed foods. It should be mentioned that food manufacturers can use GF labelling also as a marketing tool to attract consumers, but when perceiving such free-from labelled product as healthier, consumers can oversee the possible poorer nutritional composition of these products. A similar problem was highlighted also for other types of attractive claims and marketing strategies, that are used on foods [6,41,42]. It is necessary to encourage celiac patients and those following a GF diet to consume more whole GF foods, such as legumes, fruits, vegetables and whole grain GF cereals, for example buckwheat, millet and quinoa, which offer a range of nutritional benefits, in comparison with processed GF foods. GF alternatives are also maize and rice which should be encouraged to be consumed in a non-processed, wholegrain state, maintaining more fibre and beneficial nutrients. Those avoiding gluten containing foods should therefore not only rely on a range of GF processed foods but rather look for whole food alternatives, which is advisable for the general population as well. This also applies for those who are voluntarily following a GF diet. On other hand, replacing processed foods with processed GF alternatives is not likely to bring any health benefits to people without medical predispositions requiring a GF diet and could be even associated with higher cost [17,43]. There is increasing evidence that individuals who must strictly follow a GF diet are at risk for having a poorer diet and nutritional status due to lower quantities of beneficial nutrients, such as protein, fibre and micronutrients [11,44,45] and relying on processed GF foods could further contribute to this. The nutrition of CD patients should be carefully planned to ensure adequate intake of all macro- and micronutrients, especially dietary fibre, protein, as well as certain vitamins and minerals. It is also suggested that a clinical dietitian would be a part of the medical team treating CD patients [12]. If CD patients mainly rely on processed GF foods, they do not only risk malnutrition but also other possible health problems, such as obesity and various non-communicable chronic diseases associated with excessive consumption of processed foods, which are often high in fat, salt and sugar. Including whole, unprocessed foods in daily nutrition would increase the fibre and micronutrient intake of CD patients. This applies also to healthy people, who follow a GF diet by choice. By consuming GF processed foods, they are at risk of lower fibre and protein intake as well as the intake of certain micronutrients. As we have shown in our study, GF foods in general do not have better nutritional composition than regular foods. With excessive consumption of processed GF foods healthy people would not improve their nutrition but rather consume more products which are generally advised to be consumed in minimal amounts. People without medical problems related to gluten should be more informed about healthy choices and gluten in general. Some people might feel discomfort when consuming certain foods, but this is often not really related to the gluten content of foods but rather to some other carbohydrate components, such as fermentable oligo-, di-, mono-saccharides and polyols (FODMAP) which are often present in foods that contain gluten too [3]. Regarding the fact that processed GF foods often consist of refined flour and starch which do not contain numerous beneficial nutrients like wholegrain or other cereal ingredients would, it is a disadvantage for healthy people to replace possible more nutritious foods with such products, risking obesity and other health problems related with excess consumption of highly processed GF foods.

It would be useful for future studies to investigate the vitamin and mineral contents of packed GF foods, as well as the dietary patterns of people adhering to such a diet. A challenge for food manufacturers could be considering fortification of GF foods to achieve improved nutritional quality of processed GF foods. Fortification of GF foods could also compensate for possible micronutrient deficiency in CD patients [46]. A key strength of our study is in a very large dataset, collected from all the major food stores, making it very relevant indicator for the Slovenian food supply. A large sample including a range of food categories, makes the results comparable with studies conducted in other countries, and providing good insights into the GF food market. Considering that the Slovenian food supply is embedded in the European Union food market and that our sample included major international food brands, the study results are internationally relevant. A limitation of the study is that quality assessment was done using food labelling data, and not using a laboratory analyses of those foods. This means that we relied on the data provided by food manufacturers. Another study limitation is that the content of some food constituents was not always labelled; nutritional composition of foods in such cases was estimated with data from food composition databases. This disabled direct comparison of certain nutrients among GF-labelled and regular products on the whole sample. This was particularly the case with dietary fibre, for which food labelling is not mandatory in the European Union. In our sample, data on amount of saturated fats were also incomplete for some products so this nutrient was not included in the specific nutrient comparison. In future studies it would be interesting to include such nutrients as it is known that processed foods can contain excessive amounts of certain fats. To study nutritional composition in more detail, the use of specific types of fats (e.g., palm oil, hydrogenated fats, etc.) could be also considered.

## 5. Conclusions

The assessment of nutritional quality of GF-labelled foods showed that in most cases such foods cannot be considered as nutritionally superior compared to foods not labelled as gluten-free. Considering this, it is not likely that a GF diet would bring any health benefits to individuals without medical predispositions for following a GF diet. Those for whom a GF diet is required should plan their diets carefully in order to provide a balanced diet and try to avoid frequent consumption of processed GF foods. GF foods were often found to be lower in protein and consuming whole GF cereals and bakery products, that are richer in protein, fibre and beneficial nutrients in comparison to processed GF products would be a better alternative. From this perspective, public health initiatives should aim towards promoting whole food GF diets for those who are required to follow them due to medical reasons, and to encourage individuals to consume more unprocessed foods. Efforts should also be put into informing consumers about gluten, its function in food products, influence on health and evidence-based reasons for avoidance of gluten-containing foods.

## Figures and Tables

**Table 1:** Gluten-free (GF) labelled cereal products and comparison of content of energy and certain nutrients in GF-labelled vs. regular products for selected food categories.

Food Category	*N*	GF (%)	Energy (kJ/100 g)			Sugar (g/100 g)			Salt (g/100 g)			Protein (g/100 g)		
			Mean	SD	*P*	Mean	SD	*P*	Mean	SD	*P*	Mean	SD	*P*
**Bread and Bakery Products**														
Biscuits (*N* = 463)														
GF	30	6	1998.6	164.5	ns	26.9	9.6	0.22	0.6	0.4	ns	4.6	1.9	<0.01
Regular	430	94	1996.0	226.2		30.4	12.5		0.5	0.6		6.7	2.2	
Mean diff.			2.6			−3.5			0.0			−2.1		
Bread (*N* = 275)														
GF	26	9	1260.2	320.7	ns	3.2	2.9	1.00	1.3	0.4	ns	5.0	2.6	<0.01
Regular	249	91	1368.8	321.1		3.1	2.3		1.4	0.5		9.7	2.9	
Mean diff.			−108.6			0.1			−0.1			−4.8		
Cakes, muffins and pastry (*N* = 233)														
GF	28	12	1692.5	266.9	ns	15.4	15.3	<0.01	0.4	0.3	0.01	6.7	1.7	0.01
Regular	205	82	1612.5	342.6		25.4	13.4		0.6	0.4		6.0	2.3	
Mean diff.			80.0			−10.0			−0.2			0.7		
**Cereal and Cereal Products**														
Breakfast cereals (*N* = 274)														
GF	20	7	1667.1	147.3	ns	18.9	11.9	0.76	0.8	0.6	0.03	8.3	2.6	ns
Regular	254	93	1632.3	157.6		18.1	11.4		0.5	0.6		9.6	3.5	
Mean diff.			34.7			0.8			0.4			−1.3		
Pasta														
Dry pasta (*N* = 287)														
GF	28	10	1333.9	461.3	<0.011	0.8	1.0	<0.01	0.1	0.3	ns	6.3	2.7	<0.01
Regular	259	90	1485.4	151.0		2.9	1.0		0.1	0.3		13.0	1.8	
Mean diff.			−151.4			−2.2			0.0			−6.6		

*N*—number of all products; SD—standard deviation; Mean diff.—mean difference; ns–non-significant.

**Table 2:** Gluten-free (GF) labelled non-cereal products and the comparison of energy content and certain nutrients in GF-labelled vs. regular products for selected food categories.

Food Category	*N*	GF (%)	Energy (kJ/100 g)			Sugar (g/100 g)			Salt (g/100 g)			Protein (g/100 g)		
			Mean	SD	*P*	Mean	SD	*P*	Mean	SD	*P*	Mean	SD	*P*
**Meat and Meat Products**														
Processed meat and derivatives (*N* = 464)														
GF	67	14	972.5	575.5	0.04	0.6	0.5	ns	2.3	1.2	ns	17.1	6.3	ns
Regular	397	86	1111.2	496.5		0.5	0.4		2.4	1.5		17.4	7.2	
Mean diff.			−138.7			0.1			0.0			−0.3		
Sauces and spreads (*N* = 366)														
GF	39	11	1181.4	759.0	ns	13.8	17.8	ns	1.6	1.3	ns	4.7	4.6	ns
Regular	327	89	1132.0	948.5		11.6	16.8		2.2	3.2		3.9	4.5	
Mean diff.			49.3			2.1			−0.6			0.8		
**Dairy**														
**Milk**														
Milk imitates (*N* = 89)														
GF	58	65	240.8	67.9	ns	5.2	2.7	ns	0.1	0.1	1.00	1.7	1.5	ns
Regular	31	35	217.9	59.7		4.2	2.2		0.1	0.0		1.8	1.4	
Mean diff.			22.9			0.9			0.0			−0.1		
**Fish and Fish Products**														
Canned fish and seafood (*N* = 157)														
GF	30	19	898.2	279.9	ns	1.9	1.9	<0.01	1.1	0.3	ns	15.4	5.7	<0.01
Regular	127	81	988.6	413.1		0.9	1.6		1.4	1.6		17.4	6.3	
Mean diff.			−90.5			1.1			−0.3			−2.0		

*N*—number of all products; SD—standard deviation; Mean diff.—mean difference; ns—non-significant.

## Supplementary Materials

The following are available online at https://www.mdpi.com/1660-4601/17/21/8239/s1, Supplementary Table S1: Gluten-free labelling and results of nutrient profiling using NPSC per category.
