# Supplementary material for: Nutritional Composition of Gluten-Free Labelled Foods in the Slovenian Food Supply
Source: Int J Environ Res Public Health. 2020 Nov 7;17(21):8239. doi: 10.3390/ijerph17218239 (PMC7664664; doi:10.3390/ijerph17218239)
Supplement: Supplementary file 1 [file ijerph-17-08239-s001.pdf]

International Journal of
Environmental Research and Public Health

MDPI

Supplementary material

Table S1. Gluten-free labelling and results of nutrient profiling using NPSC per category (GF – gluten-free).

	All products (N)	GF (N)	GF (%)	»Healthier« (N)	»Less healthy« (N)
**Beverages**	922	10	1	10	
**Coffee and tea**	156	1	1	1	
Coffee mixes	90				
Instant coffee	10				
Tea	56	1	2	1	
**Cordials**	74				
Cordials	74				
**Electrolyte drinks**	14				
Electrolyte drinks	14				
**Fruit and vegetable juices**	254	8	3	8	
Juices	149	8	5	8	
Nectars	105				
**Soft drinks**	366	1	0	1	
Energy drinks	25				
Soft drinks	341	1	0	1	
**Waters**	58				
Waters	58				
**Bread and bakery products**	971	84	9	30	54
**Biscuits**	463	30	6		30
Biscuits	463	30	6		30
**Bread**	275	26	9	16	10
Bread	127	17	13	13	4
Crispy bread	96	7	7	2	5
Dough	39	1	3		1
Tortillas	13	1	8	1	
**Cakes, muffins and pastry**	233	28	12	14	14
Cakes, muffins & pastry	233	28	12	14	14
**Cereal and cereal products**	897	72	8	57	15
**Breakfast cereals**	274	20	7	8	12
Breakfast cereals	240	20	8	8	12
Cereal flakes and bran	34				
**Cereal bars**	46				
Cereal bars	46				
**Pasta**	422	34	8	31	3
Dry filled pasta	4				
Dry pasta	287	28	10	27	1
Fresh filled pasta	67	3	4	1	2
Fresh pasta	2				
Noodles	62	3	5	3	
**Rice**	96	11	11	11	
Rice	96	11	11	11	
**Unprocessed cereals**	59	7	12	7	
Unprocessed cereals	59	7	12	7	
**Confectionery**	968	30	3		30
**Chewing gum**	57	1	2		1
Chewing gum	57	1	2		1
**Chocolate and sweets**	828	23	3		23
Chocolate and sweets	828	23	3		23
**Jelly**	83	6	7		6
Jelly	83	6	7		6
**Convenience foods**	566	18	3	10	8
**Other**	128	5	4	5	
Side dishes	128	5	4	5	
**Pizza**	31	1	3	1	

	All products (N)	GF (N)	GF (%)	»Healthier« (N)	»Less healthy« (N)
Pizza	31	1	3	1	
**Pre-prepared salads and sandwiches**	61	1	2	1	
Pre-prepared salads and sandwiches	61	1	2	1	
**Ready meals**	180	3	2	2	1
Ready meals	180	3	2	2	1
**Soup**	166	8	5	1	7
Soup	166	8	5	1	7
**Dairy**	1430	123	9	75	48
**Cheese**	358	6	2	3	3
Cheese	195	3	2	3	
Cheese imitates	2	2	100		2
Cottage cheese	42				
Spreads and processed cheese	119	1	1		1
**Cream**	97	7	7	4	3
Cream	71				
Cream imitates	26	7	27	4	3
**Desserts**	117	22	19	20	2
Desserts	117	22	19	20	2
**Ice cream and edible ices**	190	14	7		14
Ice cream and edible ices	190	14	7		14
**Milk**	202	60	30	35	25
Instant cocoa	30	1	3	1	
Milk	61				
Milk drinks	22	1	5	1	
Milk imitates	89	58	65	33	25
**Yoghurt products**	466	14	3	13	1
Flavoured yogurt	243	3	1	2	1
Flavoured yogurt drinks	87				
Plain yogurt	110				
Yogurt imitates	26	11	42	11	
**Edible oils and emulsions**	239				
**Butter and margarine**	80				
Butter	29				
Margarine	51				
**Cooking oils**	159				
Cooking oils	159				
**Eggs**	22				
**Eggs**	22				
Eggs	22				
**Fish and fish products**	227	30	13	19	11
**Canned fish and seafood**	157	30	19	19	11
Canned fish	90	10	11	2	8
Canned fish with vegetable	31	13	42	10	3
Fish spreads	36	7	19	7	
**Chilled fish**	70				
Processed fish products	25				
Unprocessed fish	45				
**Fruit and vegetables**	818	17	2	12	5
**Fruit**	201	7	3	6	1
Canned fruit	69	1	1	1	
Dried fruit	94	5	5	4	1
Frozen fruit	18				
Nuts and fruit mixes	20	1	5	1	
**Jam and spreads**	122	7	6	3	4
Jam and spreads	122	7	6	3	4
**Nuts and seeds**	108	2	2	2	
Nuts and seeds	108	2	2	2	
**Vegetables**	387	1	0	1	
Canned vegetables	293	1	0	1	
Dried vegetables	11				
Frozen vegetables	83				
**Meat and meat products**	510	68	13	14	54

	All products (N)	GF (N)	GF (%)	»Healthier« (N)	»Less healthy« (N)
**Meat alternatives**	46	1	2	1	
Meat alternatives	46	1	2	1	
**Processed meat and derivatives**	464	67	14	13	54
Animal fat products	11	2	18		2
Meat spreads	66	7	11		7
Processed meat	348	57	16	12	45
Unprocessed meat	39	1	3	1	
**Sauces and spreads**	366	39	11	9	30
**Mayonnaise/dressings**	53	7	13		7
Mayonnaise/dressings	53	7	13		7
**Sauces**	220	14	6	6	8
Other souces	135	6	4	1	5
Pasta sauces	79	8	10	5	3
Sweet sauces	6				
**Spreads**	93	18	19	3	15
Nut spreads	17	2	12		2
Other spreads	2				
Sweet spreads	35	7	20		7
Vegetable spreads	39	9	23	3	6
**Snackfoods**	231	18	8	4	14
**Crisps and snacks**	231	18	8	4	14
Crisps and snacks	231	18	8	4	14
